# Association of lumbar disc degeneration with low back pain in middle age in the Northern Finland Birth Cohort 1966

**DOI:** 10.1186/s12891-022-05302-z

**Published:** 2022-04-15

**Authors:** Teija Mertimo, Jaro Karppinen, Jaakko Niinimäki, Roberto Blanco, Juhani Määttä, Markku Kankaanpää, Petteri Oura

**Affiliations:** 1 Faculty of Medicine and Health Technology, Tampere University Hospital and University of Tampere, P.O. Box 607, FI-33014 Tampere, Finland; 2 Center for Life Course Health Research, Faculty of Medicine, University of Oulu, P.O. Box 5000, FI-90014 Oulu, Finland; 3 Medical Research Center Oulu, Oulu University Hospital and University of Oulu, P.O. Box 5000, FI-90014 Oulu, Finland; 4 Rehabilitation Services of South Karelia Social and Health Care District, Lappeenranta, Finland; 5 Research Unit of Medical Imaging, Physics and Technology, Faculty of Medicine, University of Oulu, P.O. Box 5000, FI-90014 Oulu, Finland; 6 Department of Radiology, Turku University Hospital, Kiinamyllynkatu 4-8, FI-20520 Turku, Finland; 7 Department of Rehabilitation and Psychosocial Support, Tampere University Hospital, P.O. Box 2000, FI-33521 Tampere, Finland

**Keywords:** Lumbar disc degeneration, Low back pain, Magnetic resonance imaging, Back-related functional limitation, Mental distress, Prevalence, Finland, Cohort studies

## Abstract

**Background:**

Although it has been suggested that lumbar disc degeneration (LDD) is a significant risk factor for low back pain (LBP), its role remains uncertain. Our objective was to clarify the association between LDD and LBP and whether mental distress modifies the association.

**Methods:**

Participants of a birth cohort underwent 1.5-T lumbar magnetic resonance imaging at the age of 47. The association between the sum score of LDD (Pfirrmann classification, range 0–15) and LBP (categorized into “no pain”, “mild-to-moderate pain”, “bothersome-and-frequent pain”) was assessed using logistic regression analysis, with sex, smoking, body mass index, physical activity, occupational exposure, education, and presence of Modic changes and disc herniations as confounders. The modifying role of mental distress (according to the Hopkins Symptom Check List-25 [HSCL-25], the Beck Depression Inventory and the Generalized Anxiety Disorder Scale) in the association was analyzed using linear regression.

**Results:**

Of the study population (*n* = 1505), 15.2% had bothersome and frequent LBP, and 29.0% had no LBP. A higher LDD sum score increased the odds of belonging to the “mild-to-moderate pain” category (adjusted OR corresponding to an increase of one point in the LDD sum score 1.11, 95% CI 1.04–1.18, *P* = 0.003) and the “bothersome-and-frequent pain” category (adjusted OR 1.20, 95% CI 1.10–1.31, *P* < 0.001), relative to the “no pain” category. Mental distress significantly modified the association between LDD and LBP, as a linear positive association was consistently observed among individuals without mental distress according to HSCL-25 (adjusted B 0.16, 95% CI 0.07–0.26, *P* < 0.001), but not among individuals with higher mental distress.

**Conclusions:**

LDD was significantly associated with both mild-to-moderate and bothersome-and-frequent LBP. However, the co-occurrence of mental distress diminished the association between LDD and LBP bothersomeness. Our results strongly suggest that mental symptoms affect the pain experience.

**Supplementary Information:**

The online version contains supplementary material available at 10.1186/s12891-022-05302-z﻿.

## Background

Low back pain (LBP) is a common health problem and causes more disability than any other health problem worldwide [1–3]. The prevalence of disabling LBP grew significantly between 1990 and 2015 [3], and LBP-related disability and economic burden are expected to further increase in the decades to come [3]. Although LBP has a few specific causes such as vertebral fracture, malignancy or infection, approximately 90% of LBP is considered nonspecific, i.e., the cause of pain cannot be identified [3, 4].

Lumbar disc degeneration (LDD) is very prevalent among middle-aged populations [5]. Evidence from twin [5, 6] and cohort studies [7–13] suggest that LDD is a significant risk factor for LBP. However, there are also several studies that have not found evidence for an association between LDD and LBP [14, 15]. Furthermore, radiological features of LDD are also frequent among asymptomatic individuals [16, 17], and presence of LDD appears not to predict future LBP [15, 18]. These contradicting findings on the association between LDD and LBP perplex clinicians evaluating the source of pain among their patients as they cannot be sure of the source of pain among their patients with LDD and LBP.

The presence and severity of LBP are closely related to a wide range of biophysical, psychological, and social elements [3]. Importantly, it has been suggested that psychosocial factors, such as symptoms of depression and anxiety, contribute to the chronification of pain [3, 19, 20]. Pain can persist even if the original tissue source of pain has healed [21].

In the vast majority of the people the cause of back pain is unknown and disability caused by LBP is high in working age groups [3]. Many studies have shown the association between LDD and LBP [5, 6]. However, it has been suggested that the LBP experience is not necessarily explained purely by structural findings such as LDD in MRI [15]. It is important to gain more knowledge of the factors that are associated with nonspecific LBP. Mental distress is suggested to increase the risk of disabling LBP [3]. However, the role of mental distress in the association between LDD and LBP has been poorly studied. We speculate that the contradicting findings of previous studies on the role of LDD in LBP could be partly explained by the modifying role of mental distress. From a clinical perspective it would be essential to know whether mental distress, as revealed by a questionnaire, may be involved in the association between LDD and LBP. This knowledge could then be used to achieve a treatment strategy that leads to a successful outcome.

## Methods

### Aims

Our aims were to evaluate the association between LDD and LBP, and clarify whether mental distress modifies the association between LDD and the bothersomeness of LBP among individuals with pain, in a large unselected general population sample of Northern Finns who had undergone magnetic resonance imaging (MRI) of the lumbar spine in midlife.

### Study design

A retrospective cross-sectional study using prospectively collected data.

### Study sample

The Northern Finland Birth Cohort 1966 (NFBC1966) is a prospective longitudinal population-based cohort study (*n* = 12 058 live births) comprising inhabitants of the two northernmost provinces of Finland (Oulu and Lapland). Pregnant women whose expected date of delivery was between January 1st and December 31st, 1966 were invited to participate in the cohort study. The cohort participants and their mothers have been followed since 1965–1966 via regular postal questionnaires, clinical examinations, and data collected from health care records [22, 23].

In this cross-sectional study, we used data from the most recent follow-up at the age of 47 in 2012–2014. We used cross-sectional data from the most recent follow-up as the MRI was obtained only at this time point. In 2012–2014, the participants first filled in questionnaires, which were followed by clinical examinations and MRI. Postal questionnaires were sent to all the individuals whose addresses were known to gather information on their health status, socioeconomic status, and lifestyle habits. The response rate was 66.5% (*n* = 6868, target population 10,321). Cohort members who lived in Finland were asked to undergo clinical examinations, and 5861 (57%) individuals participated.

The individuals who attended the clinical examinations and were living within 100 km of the city of Oulu (*n* = 1988) were invited to undergo lumbar MRI at the Oulu University Hospital. Of those invited, 448 clinical examination participants did not undergo MRI due to 1) not showing up, 2) claustrophobia, 3) severe obesity preventing the use of the imaging equipment, or 4) a pacemaker. A total of 1540 underwent MRI. After excluding participants with missing images and other technical errors, we performed LDD consensus readings for 1505 individuals. Those with missing background data were excluded (*n* = 202) from the further analyses and thus the final sample for analyses of pain consisted of 1303 participants.

### Assessment of lumbar MRI and evaluation of LDD

To obtain the lumbar MR images we used 1.5-T MRI (Signa HDxt, General Electric, Milwaukee, WI) in 2012–2014, when the participants were aged 47. The MRI was transacted by T2-weighted fast-recovery fast spin-echo (frFSE) images in the sagittal (repetition time/effective echo time (TR/effTE) 3500/112 ms, 4 averages, field-of-view (FOV) 280 × 280 mm, and the acquisition matrix was 448 × 224, slice thickness 3 mm with 1 mm interslice gap) and transverse planes (TR/effTE 3600/118 ms, 4 averages, FOV 180 × 180 mm, acquisition matrix 256 × 224, slice thickness 4 mm with 1 mm interslice gap) and T1-weighed fluid-attenuated inversion recovery sequence images in sagittal plane (TR/effTE 860/20 ms, inversion time (TI) of 1969 ms, 1.5 averages, FOV 280 × 280 mm, acquisition matrix 256 × 224, slice thickness 4 mm, interslice gap 1 mm). The scans were evaluated using NeaView Radiology software (Neagen Oy, Oulu, Finland), version 2.31.

We used the Pfirrmann classification [24] to assess LDD: Grade I (normal shape, no horizontal bands, clear distinction of the nucleus and annulus), Grade II (non-homogeneous shape with horizontal bands, some blurring between the nucleus and annulus), Grade III (non-homogeneous shape with blurring between the nucleus and annulus, annulus shape still recognizable), Grade IV (non-homogeneous shape with hypointensity, annulus shape not intact and distinction between the nucleus and annulus impossible, disc height usually decreased), and Grade V, which was the same as Grade IV but with a collapsed disc space.

First, two experienced musculoskeletal radiologists (JN and RB) and one experienced physiatrist with a strong history in spinal imaging (JK) independently assessed the lumbar MRI images. Then, the first author (TM) pursued a consensus. All evaluators were blinded to the other data and parameters used in the study.

JK evaluated all 1505 images in random order. JN also evaluated 826 of these images and RB evaluated 753. Although the differences between the readers were generally small, TM carefully re-evaluated all the discs with discordant grading (i.e., difference of one or more Pfirrmann grades between the original evaluators), consulting JN and JK when needed.

Once the LDD consensus was reached, the overall burden of LDD was quantified by constructing a sum score variable, as described previously [7]. To calculate the LDD sum score, the degree of degeneration at each lumbar level was summed on the basis of the Pfirrmann classification by categorizing grades I and II as 0, and grades III, IV and V as 1, 2 and 3 respectively. Thus, the LDD sum score for five lumbar discs could theoretically range from 0 to 15, with higher values indicating higher overall LDD [7]. In order to obtain a balanced sum score for each individual, we used the case-median method to impute the Pfirrmann grades for the few discs with missing values (17 out of the 7525 discs, 0.2%) [25].

### Assessment of low back pain and other musculoskeletal pains

Data on the presence, bothersomeness and frequency of LBP and other musculoskeletal pains were collected using a questionnaire issued to the participants at the time of the lumbar MRI. The anatomical area of pain sites was illustrated by a drawing. Left and right sides were elicited separately (neck, low back, shoulders, elbows, wrists and hands, hips, knees, ankles, and feet). LBP and other pain sites were elicited using the following questions: 1) “Have you had any aches or pains in your lower back within the last 12 months? (no / yes)”. If the answer was positive, the next question was 2) “How often have you had aches or pains during the last 12 months? (1–7 days, 8–30 days, > 30 days, daily)”. If the respondent had experienced LBP during the last 12 months, we asked them about the pain’s total bothersomeness during work, leisure time and sleep (altogether), rating it on a numerical rating scale (NRS) from 0 (no pain) to 10 (extremely bothersome pain).

The participants were divided into three LBP categories. The “no pain” category contained individuals who reported no LBP during the previous 12 months. In accordance with a previous study in the same population [26], “bothersome-and-frequent pain” was defined as pain bothersomeness of ≥6 lasting over 30 days during the last 12 months. The cut-off of ≥6 for bothersomeness was also supported by the distribution of the present data, as it formed the threshold for the highest tertile of bothersomeness. The “mild-to-moderate pain” category was defined as pain bothersomeness of < 6 or pain lasting under 30 days during the last 12 months.

When analyzing the role of mental distress in the association between LDD and total bothersomeness of LBP among individuals who reported any LBP, we modelled bothersomeness (theoretical range 0–10) as a continuous variable. To assess “multiple pain sites”, we divided the participants into two categories, depending on whether they had experienced more than, or less than 30 days of pain during the last 12 months, in more than one location. Neck and low back were each considered one pain site, regardless of whether the pain was left or right sided. Those with ≥2 pain sites were considered to have “multiple pain sites”.

### Assessment of mental distress

As part of the follow-up questionnaires at the age of 47, we asked the respondents to fill in the Hopkins Symptom Check List-25 [27] (HSCL-25), the Generalized Anxiety Disorder 7-item Scale [28] (GAD-7) and the Beck Depression Inventory [29] (BDI-21), which elicit mental distress, i.e., symptoms of depression and anxiety. The cut-off point for clinically relevant mental distress in the HSCL-25 was set at 1.55, which has been used before [30, 31]. This relatively low cut-off point was selected as it has been used in previous NFBC1966 studies and it ensured sufficient group sizes for further analyses. For the BDI-21, the cut-off point was between normal (0–12 points) and mild depression (13–18 points) [32, 33], whereas for the GAD-7, the cut-off point was between normal (0–4) and mild anxiety (5–9 points) [28, 34]. These relatively low cut-off points were selected in order to ensure sufficient group sizes for further analyses and because they are the Finnish guidelines’ cut-off points for mild symptoms of depression and anxiety.

### Assessment of confounders

Based on previous studies, sex, smoking, body mass index (BMI), leisure-time physical activity, occupational physical exposure, education, and Modic changes and disc herniations presenting in lumbar MRI were considered to be potential confounders in the association between LDD and LBP [2, 3, 35–48]. These variables were recorded at the 47-year follow-up.

In the clinical examination, a trained nurse measured the height and weight of each participant. BMI was calculated as kilograms per meter squared and categorized according to the World Health Organization (WHO) definition (normal weight: BMI < 25, overweight: BMI 25–30, and obesity: BMI > 30) [49].

The level of education was determined as years of study: < 9 school years, 9–12 school years, > 12 school years. This classification was also used in a previous study [26] and is based on the Finnish education system.

Smoking was elicited by two questions: 1) “Have you ever smoked cigarettes (yes/no)?” and 2) “Do you currently smoke (yes/no)?” Based on the answers, the participants were classified into three groups: 1) never-smokers, 2) former smokers and 3) current smokers. This variable has also been used in a previous study [26].

When assessing physical activity during leisure time, we used a previously introduced variable [41]. We asked the participants how often they took part in physical activity causing at least some sweating and breathlessness, corresponding to moderate-to-vigorous intensity. The six response alternatives were 1) daily, 2) 4–6 times a week, 3) 2–3 times a week, 4) once a week, 5) 2–3 times a month, and 6) once a month or less often. The individuals were divided into three categories: “active” (at least 4 times a week), “moderately active” (1–3 times a week), and “inactive” (less than once a week).

Occupational physical exposure was assessed in the way described previously [43]. Individuals were classified into two groups according to their occupational physical activity: “low” (high-intensity tasks [i.e. hard physical labor, constant moving, and lifting heavy loads] performed rarely or occasionally) and “high” (at least one of the high-intensity tasks performed at least often).

We also used the presence of lumbar disc herniations and Modic changes as covariates. An experienced lumbar MRI reader (JK) evaluated the presence of disc herniations, dichotomizing them as “no disc displacement or bulge”, or “protrusion, extrusion or sequester”. Modic changes were previously evaluated by consensus reading [26] and were used here as “no” or “yes”.

### Statistical analyses

Statistical analysis was performed using SPSS Statistics, version 27, 64-bit edition (IBM, Armonk, NY, USA). The threshold of statistical significance was set at *P* = 0.05. We used descriptive statistics to present the distributions of LDD, LBP and background variables: frequencies (n) and percentages (%) for categorical variables, and means with standard deviations (SD) or medians with interquartile ranges (IQR) for continuous variables, depending on normality. The characteristics of the sample were compared to those of the excluded individuals by means of a Chi-square test and independent-samples t test for the categorical and continuous variables, respectively.

Interrater reliability in the Pfirrmann classification was evaluated using Weighted Kappa. The Kappa values were interpreted as follows: 0–0.20, poor; 0.21–0.40, fair; 0.41–0.60, moderate; 0.61–0.80, good; and 0.81–1.00, very good [50].

We modeled the association between the LDD sum score (continuous predictor) and the LBP category (three-class outcome) using multinomial logistic regression. The “no pain” category was used as the reference against which the other categories were compared. We constructed both unadjusted and adjusted models. Odds ratios (OR), their 95% confidence intervals (CI), and the corresponding *P* values were obtained from each model.

The role of mental distress (binary moderator; yes/no according to the HSCL-25, BDI-21 and GAD-7) and multiple pain sites (binary moderator; < 2 pain sites / ≥ 2 pain sites) in the association between LDD sum score (continuous predictor) and bothersomeness of LBP (continuous outcome) was analyzed using linear regression models stratified by the presence of mental distress and/or pain sites. The beta coefficient (B), its 95% CI and the corresponding P value were obtained from the stratified models. Mental distress*LDD and multiple pain sites*LDD interaction terms were also incorporated into the non-stratified models, and the statistical significance of the interaction terms was used to confirm the modifying effect.

Multicollinearity was examined by means of variance inflation factor (VIF) values. As all VIFs were ≤ 1.38, the models did not have multicollinearity issues [51].

### Ethical approval

The study followed the principles of the Declaration of Helsinki and its later amendments. The study was approved by the Ethics Committee of the Northern Ostrobothnia Hospital District in Finland. Participation in the NFBC1966 was voluntary and each study participant granted their written informed consent. Personal identity information was encrypted and pseudonymized before the data were handed over to the researchers.

## Results

### Characteristics of the study population

LDD consensus was achieved among 1505 participants who had undergone MR imaging at a mean age of 47 (SD 0.4). Table 1 shows the characteristics of the LDD consensus sample, with comparison to the rest of the NFBC1966 population. Of the present sample, 15.2% had bothersome and frequent LBP, whereas 29.0% had no LBP. Minor but statistically significant differences were found between the sample and those excluded in terms of sex distribution, smoking and education.

**Table 1:** Characteristics of the study population and comparison with rest of the NFBC1966 population. Variation of N is due to missing data

	Excluded (*n* = 4297)	Included (*n* = 1505)	*P* value for difference
	% (n) / Mean (SD)	% (n) / Mean (SD)	
Sex			**0.003**
Men	51.0 (4458)	46.8 (703)	
Women	49.0 (4286)	53.2 (799)	
Body Mass Index (kg/m^2^)	26.9 (5.0)	26.7 (4.6)	0.114
< 25	38.7 (1662)	40.9 (614)	
25–30	39.6 (1701)	38.5 (578)	
> 30	21.7 (932)	20.5 (308)	0.294
Smoking			**< 0.001**
Non-smoker	50.8 (2634)	54.0 (779)	
Former	25.9 (1344)	29.5 (426)	
Current	23.2 (1205)	16.5 (238)	
Education years			**0.016**
< 9	4.2 (221)	3.3 (48)	
9–12	68.3 (3574)	72.0 (1053)	
> 12	27.5 (1441)	24.7 (361)	
Leisure-time physical activity (times/week)			0.377
< 1	28.1 (1470)	26.5 (384)	
1–3	56.4 (2955)	56.8 (823)	
> 4	15.5 (812)	16.6 (241)	
Occupational physical exposure			0.053
Low	62.7 (3148)	59.8 (845)	
High	37.3 (1875)	40.2 (567)	
LBP category			–
Bothersome-and-frequent pain	–	15.2 (223)	
Mild-to-moderate pain	–	55.8 (820)	
No pain	–	29.0 (427)	
LDD sum score^a^ (median with IQR)	–	4 (2–6)	–
Normal discs (sum score = 0, Pfirrmann = II)	–	5.0 (75)	
At least mild degeneration (sum score ≥ 1, at least one disc Pfirrmann ≥ III)	–	95.0 (1427)	
Modic changes			–
No	–	33.0 (492)	
Yes	–	67.0 (997)	
Disc herniations			–
No disc displacement or bulge	–	80.3 (1195)	
Protrusion, extrusion or sequester	–	19.7 (294)	

^a^The severity of lumbar disc degeneration was formulated from the Pfirrmann degeneration scale by categorizing grades I and II as 0, and grades III, IV and V as 1, 2 and 3, respectively. Thus, the LDD sum score for five lumbar discs could theoretically range from 0 to 15. *CI* Indicates confidence interval; *LBP* Low back pain, *LDD* Lumbar disc degeneration, *SD* Standard deviation, *IQR* Interquartile range

### Inter-rater reliability in the Pfirrmann classification

Table 2 shows the Kappa values demonstrating inter-rater reliability in the Pfirrmann classification. The reliability between readers JK and JN (κ = 0.74 to 0.78) and readers JN and RB was good (κ = 0.61 to 0.79). The reliability between readers JK and RB ranged from fair to good (κ = 0.39 to 0.69).

**Table 2:** Inter-rater reliability in the Pfirrmann classification. Weighted Kappa values (κ) with 95% confidence intervals (95% CI) by lumbar level (*n* = 50 cases in each comparison)

Lumbar level	Reader #1 (J.K.) vs. Reader #2 (J.N.)		Reader #1 (J.K.) vs. Reader #3 (R.B.)		Reader #2 (J.N.) vs. Reader #3 (R.B.)	
	κ	95% CI	κ	95% CI	κ	95% CI
L1/L2	0.78	0.63–0.93	0.41	0.19–0.62	0.69	0.51–0.88
L2/L3	0.76	0.62–0.91	0.39	0.19–0.59	0.61	0.42–0.79
L3/L4	0.75	0.60–0.91	0.58	0.39–0.76	0.79	0.64–0.94
L4/L5	0.77	0.64–0.90	0.69	0.55–0.83	0.64	0.49–0.80
L5/S1	0.74	0.62–0.86	0.63	0.50–0.76	0.65	0.51–0.79

### Prevalence of LDD

Table 3 presents the distribution of Pfirrmann grades by lumbar level. In general, the prevalence of degenerative changes increased towards lower lumbar levels. Five percent of the participants had all discs classified as normal (Pfirrmann ≤ II) and the vast majority (95.0%) had at least one mildly degenerated disc (Pfirrmann ≥ III). The median LDD sum score was 4 (2–6) (Table 1). The prevalence of advanced LDD corresponding to at least one Pfirrmann V disc was higher in the “bothersome-and-frequent pain” group (32.3%) than in the “no pain” group (18.8%) or the “mild-to-moderate pain” group (23.3%) (*p* < 0.001).

**Table 3:** Distribution of Pfirrmann grades according to consensus reading (*n* = 1505)

Pfirrmann grade	L1/L2	L2/L3	L3/L4	L4/L5	L5/S1
	% (n)	% (n)	% (n)	% (n)	% (n)
I	0 (0)	0 (0)	0 (0)	0 (0)	0 (0)
II	75.4 (1135)	63.9 (961)	47.9 (721)	22.7 (342)	21.8 (328)
III	19.8 (298)	29.1 (438)	39.7 (598)	41.4 (623)	29.8 (449)
IV	4.3 (65)	5.8 (87)	11.0 (165)	28.3 (426)	31.4 (473)
V	0.3 (4)	1.1 (17)	1.3 (19)	7.2 (109)	16.6 (250)
N/A	0.2 (3)	0.1 (2)	0.1 (2)	0.3 (5)	0.3 (5)

*N/A* Not available

### Association between LDD and LBP

A total of 1303 participants responded to the pain questionnaire, underwent MR imaging and had available data on confounders. In the logistic regression analysis, a higher LDD sum score clearly increased the odds of belonging to the “mild-to-moderate pain” and “bothersome-and-frequent pain” categories, relative to the “no pain” category (adjusted OR 1.11–1.20 corresponding to one point in the LDD sum score, *P* ≤ 0.003) (Table 4).

**Table 4:** Odds ratios demonstrating the association between LDD sum score and LBP (*n* = 1303)

	No pain (29.5%, *n* = 384 (ref))	Mild-to-moderate pain (55.1%, *n* = 718)	Bothersome-and-frequent pain (15.4%, *n* = 201)
OR (95% CI) corresponding to one point in LDD sum score			
Unadjusted	1	1.13 (1.07–1.20), *P* < 0.001	1.24 (1.15–1.33), *P* < 0.001
Adjusted^1^	1	1.11 (1.04–1.18), *P* = 0.003	1.20 (1.10–1.31), *P* < 0.001

^1^adjusted for sex, smoking, BMI, education, leisure-time physical activity, occupational physical exposure, Modic changes, and herniations. *OR* Odds ratio, *CI* Confidence interval, *LDD* Lumbar disc degeneration, *LBP* Low back pain, *Ref* Reference category (i.e. to which the other categories were compared); Mild to moderate pain, pain bothersomeness < 6 or pain under 30 days during the last 12 months; Bothersome and frequent pain, pain bothersomeness ≥6 for over 30 days during the last 12 months

### Mental symptoms modify the association between LDD and LBP

We modeled the association between the LDD sum score and the bothersomeness of LBP among individuals who reported any LBP in the pain questionnaire, stratifying it according to mental distress and multiple pain sites (*n* = 802) (Table 5). We observed a significant positive association among those with no mental distress, but none among individuals with significant mental symptoms. If the individual had no mental symptoms according to the HSCL-25, LDD and LBP were linearly associated, regardless of whether the person had pain in one or several parts of the body. The results remained similar regardless of adjustments. Supplementary Table 1 presents the models stratified according to BDI-21 and GAD-7.

**Table 5:** Beta coefficients (B) and 95% confidence intervals (CIs) for the association between LDD score and bothersomeness of pain among individuals who reported any LBP (*n* = 802)

Stratification	Unadjusted B (95% CI)	Adjusted^1^ B (95% CI)
1. All individuals with LBP (*n* = 802)	**0.16 (0.09–0.24), * P*** **< 0.001**	**0.14 (0.05–0.22),* P*** **= 0.001**
2. Mental distress (HSCL-25)		
No (*n* = 631)	**0.19 (0.10–0.27),* P*** **< 0.001**	**0.16 (0.07–0.26),* P*** **< 0.001**
Yes (*n* = 171)	0.09 (−0.09–0.26),* P* = 0.343	0.05 (−0.15–0.25),* P* = 0.619
3. Number of pain sites		
< 2 (*n* = 401)	**0.14 (0.03–0.24),* P*** **= 0.009**	**0.11 (0.00–0.23),* P*** **= 0.050**
≥ 2 (*n* = 401)	**0.19 (0.08–0.29),* P*** **< 0.001**	**0.16 (0.03–0.28),* P*** **= 0.014**
4. Mental distress (HSCL-25) and pain sites combined		
No mental distress and < 2 pain sites (*n* = 333)	**0.17 (0.06–0.28),* P*** **= 0.002**	**0.14 (0.02–0.26),* P*** **= 0.026**
No mental distress and ≥ 2 pain sites (*n* = 298)	**0.20 (0.09–0.32),* P*** **< 0.001**	**0.18 (0.03–0.32),* P*** **= 0.016**
Mental distress and < 2 pain sites (*n* = 68)	−0.06 (−0.34–0.23), *P* = 0.691	−0.03 (−0.38–0.31),* P* = 0.841
Mental distress and ≥ 2 pain sites (*n* = 103)	0.15 (−0.08–0.38), *P* = 0.188	0.08 (−0.18–0.34),* P* = 0.540

^1^adjusted for sex, smoking, BMI, education, leisure-time physical activity, occupational physical exposure, Modic changes, and herniations. *LDD* Lumbar disc degeneration, *LBP* Low back pain, *HSCL-25* Hopkins Symptom Checklist-25

## Discussion

Our study showed that LDD is extremely prevalent in the general middle-aged Northern Finnish population. We found a significant association between LDD and LBP, but the co-occurrence of mental distress attenuated the association between LDD and LBP bothersomeness.

A typical MR image in an individual aged 47 seems to be one in which at least one disc is at least mildly degenerated. The most degenerated levels were the lowest intervertebral segments, L4/5 and L5/S1, as described previously [9, 48]. Normal intervertebral discs (Pfirrmann II) were found in 75.4% of the participants at L1/L2, but in only 21.8% at L5/S1. At L5/S1, 29.8% of the participants had mild, 31.4% had moderate, and 16.6% had severe degree of disc degeneration.

We found a significant association between LDD and LBP. This finding is in line with those of previous studies and a meta-analysis [5, 6, 13]. This association was independent of other pain-related imaging findings, such as disc herniations, Modic changes, and other suggested risk factors for LBP, such as smoking, BMI, heavy physical work, leisure-time physical activity, and socioeconomic status [3]. Our study also showed that the greater the degeneration was, the greater were the odds of LBP-related bothersomeness. An increase of only one in the LDD sum score increased the odds in the “mild-to-moderate pain” group by 11% and in the “bothersome-and-frequent pain” group by 20%, which was defined in the questionnaire as combined significant decrease in physical ability at work, during leisure time and sleep lasting over 30 days during the previous 12 months. These results support those of previous research that LBP is at least partly due to LDD.

Although our study and several others have suggested that LDD is a significant risk factor for LBP [3, 52–55], there is debate over whether LDD simply occurs as a natural part of aging and is not the cause of LBP as it also occurs in asymptomatic individuals [16]. However, LDD was clearly more prevalent among the symptomatic than the asymptomatic adults in our study, and as shown previously [13]. It has been suggested that several factors can protect against pain, for example exercise and biopsychosocial education [56], even in cases of severe degeneration. In all, our results suggest that even if there is a clear association between LDD and LBP, individuals with even quite high LDD burden are not automatically destined to live with chronic and disabling pain. From this perspective, it would be interesting to determine whether a certain degree of degeneration causes pain to almost everyone. It would also be interesting to shed light on the protective factors that explain why some people with significant LDD do not suffer from recurrent and bothersome LBP.

Our second aim was to investigate how mental distress affects the association between LDD and bothersomeness of LBP. We speculate whether co-occurring mental distress could explain previous contradictory findings regarding the association between LDD and bothersomeness of LBP. We found that mental distress had a significant modifying effect on the association. The HSCL-25, BDI-21 and GAD-7 gave similar results, as their scores were each found to modify the association between LDD and LBP. If the participant did not report relevant mental symptoms, a linear association between LDD and bothersomeness of LBP was evident, suggesting that in this subgroup, the cause of LBP was primarily LDD. However, among those who reported at least mild but clinically relevant symptoms of anxiety and depression, LDD was not associated with the bothersomeness of LBP. Investigation of the association between LDD and bothersomeness of LBP stratified by mental distress and multiple pain sites revealed that mental distress was a stronger effect modifier than the presence of multiple pain sites.

LBP symptoms tend to improve quickly. Nonetheless, recurrence of LBP is common, and in some cases, the pain becomes frequently recurring or chronic [3]. The pain experience is affected by a number of factors [3]. In addition to potential psychological risk factors [3] and the factors that we considered as confounders, it has been suggested that central sensitization and memory of pain are associated with chronic pain [21]. An association between central sensitization and LBP has been proposed [57]. Individuals with co-occurring mental distress and LBP are a heterogeneous group with a variety of causes of LBP. Due to the cross-sectional nature of the our study, we do not know which leads to which, LBP to mental distress or mental distress to LBP. Nevertheless, our study suggests that the association between LDD and LBP bothersomeness is present when mental distress is not involved. Thus, although further research is needed, our results suggest that mental distress affects the pain experience.

The strengths of this study were manifold. We used an unselected sample of working-aged people and a representative MRI subsample. Our sample size was relatively large, and the measurements were reliable. Adjustments were comprehensive, making the results more reliable and minimizing the effects of confounders. The imaging area was the entire lumbar spine, and the imaging technique was standardized. The images were interpreted by expert radiologists and a physiatrist, with an external evaluator to achieve a consensus. The MR images were evaluated by the widely used Pfirrmann classification. The intra-rater reliability of LDD scoring was high. The presence of LBP was determined by questionnaires at the same time as the MRI. We asked the participants about the different dimensions of pain and the factors that affected their pain experience. Based on the analysis of representativeness, bias due to withdrawals was low. We found only a few minor differences between the sample and those excluded, implying that our findings are potentially generalizable to the Northern Finnish population.

Our study also had limitations. We believe that the main ones apply to evaluating MRI and the definition of pain. The MR images were evaluated visually, and not, for example, using automated reading of MRI scans. However, the images were evaluated by several experienced evaluators with generally high inter-rater reliability. We calculated LDD sum score in accordance with a previously published scoring system [7], adding Pfirrmann degeneration scores at different levels to arrive at a composite score. Each level and grade were equally weighted so that a Pfirrmann III degeneration at three lumbar received the same score as a Pfirrmann V degeneration at one level, for example. The definition of clinically significant pain is not universal and, therefore, in this study, the definition was chosen not only on the basis of previous literature but also on the basis of the distribution of the present data [58]. Pain categories were defined by means of frequency and bothersomeness. We preferred to use bothersomeness rather than intensity as the primary pain dimension because it was perceived as a wider concept, capturing pain-related dysfunction in work ability, leisure time and sleep. The definition of mental distress is also a limitation of this study. The individuals were divided into two groups with binary cut-offs. However, clinical use in Finland and previous studies have shown that these cut-offs distinguish well between symptomatic and asymptomatic individuals [28, 30, 32]. As we did not have detailed data on comorbidities, future studies should account for pain-related conditions such as rheumatoid arthritis as potential covariates. The analysis of representativeness showed that the present sample had small but statistically significant differences compared to the rest of the cohort. Previous studies of this subsample have noted these differences and concluded that their significance is minor [59]. As this is a cross-sectional study, the causality or longitudinal development of the MRI findings cannot be determined. LBP was elicited at a single time point but the questionnaire covered symptoms over the preceding year, whereas the HSCL-25, BDI-21 and GAD-7 measured mental symptoms over the previous weeks. The data were collected over a period of 2 years, with an individual schedule for each participant.

## Conclusions

In conclusion, our study confirmed a significant association between LDD and LBP among a general population sample of middle-aged Finns. The higher the LDD sum score, the more likely it was that pain was experienced. We also discovered that mental distress modified the association between LDD and bothersomeness of LBP; if mental distress was not present, the association between LDD and LBP existed, but if mental distress was present, the association between LDD and LBP was lost. These aspects suggest it would be crucial to clarify whether treatment guidelines need to be updated to pay mental distress even more attention in the treatment and rehabilitation of bothersome LBP. Further studies are recommended to confirm these findings and to determine whether they should be noted when diagnosing and planning the treatment and rehabilitation of individuals with LBP.

## Supplementary Information

**Additional file 1.**

## Data Availability

NFBC data is available from the University of Oulu, Infrastructure for Population Studies. Permission to use the data can be applied for research purposes via electronic material request portal. In the use of data, we follow the EU general data protection regulation (679/2016) and Finnish Data Protection Act. The use of personal data is based on cohort participant’s written informed consent at his/her latest follow-up study, which may cause limitations to its use. Please, contact NFBC project center (NFBCprojectcenter@oulu.fi) and visit the cohort website (www.oulu.fi/nfbc) for more information.

## Abbreviations

B: Beta coefficient
BDI-21: The Beck Depression Inventory
BMI: Body mass index
Bothersomeness: Bothersomeness of low back pain during work, leisure time and sleep (altogether), rating it on a numerical rating scale (NRS) from 0 (no pain) to 10 (extremely bothersome pain)
Bothersome and frequent pain: Pain bothersomeness ≥6 for over 30 days during the last 12 months
CI: Confidence interval
GAD-7: The Generalized Anxiety Disorder 7-item Scale
HSCL-25: Hopkins Symptom Checklist-25
IQR: Interquartile range
κ: Kappa value
LBP: Low back pain
LDD: Lumbar disc degeneration
Mental distress: HSCL score ≥1.55
Mild to moderate pain: Bothersomeness of low back pain < 6 or pain under 30 days during the last 12 months
MRI: Magnetic resonance imaging
NFBC1966: The Northern Finland Birth Cohort 1966
n: Number
N/A: Not available
OR: Odds ratio
Ref: Reference category
SD: Standard deviation
