# Supplementary material for: Association of lumbar disc degeneration with low back pain in middle age in the Northern Finland Birth Cohort 1966
Source: BMC Musculoskelet Disord. 2022 Apr 15;23:359. doi: 10.1186/s12891-022-05302-z (PMC9011971; doi:10.1186/s12891-022-05302-z)
Supplement: Supplementary file 1 — Additional file 1. [file 12891_2022_5302_MOESM1_ESM.docx]

SUPPLEMENTARY TABLES

**Supplementary Table 1**. Beta coefficients (B) and 95% confidence intervals (CIs) of association between LDD score and bothersomeness of pain among individuals with LBP.

| Stratification | Unadjusted B (95% CI) | Adjusted^1^ B (95% CI) |
| --- | --- | --- |
| 1. All individuals with LBP (n = 642) | **0.16 (0.08-0.24), p < 0.001** | **0.13 (0.04-0.23). p = 0.005** |
| 2. Mental distress (BDI-21) |  |  |
| No (n = 565) | **0.17 (0.09-0.26). p < 0.001** | **0.13 (0.03-0.23). p = 0.009** |
| Yes (n = 77) | 0.04 (-0.26-0.33). p = 0.810 | 0.08 (-0.25-0.41). p = 0.614 |
| 3. Mental distress (GAD-7) |  |  |
| No (n = 500) | **0.17 (0.08-0.26). p < 0.001** | **0.13 (0.03-0.23). p = 0.011** |
| Yes (n = 142) | 0.13 (-0.08-0.34). p = 0.211 | 0.06 (-0.18-0.30). p = 0.602 |

1 : adjusted for sex, smoking, BMI, education, leisure-time physical activity, occupational physical exposure, Modic changes, and herniations. LDD, Lumbar disc degeneration; LBP, Low back pain; HSCL-25; BDI-21, Beck Depression Inventory; GAD-7, General Anxiety Disorder-7.
